# Quantitative circumferential strain analysis using 3-Tesla feature-tracking cardiovascular magnetic resonance in patients with old myocardial infarction

**DOI:** 10.1186/1532-429X-18-S1-P84

**Published:** 2016-01-27

**Authors:** Ryo Ogawa, Tomoyuki Kido, Masashi Nakamura, Teruhito Kido, Akiyoshi Ogimoto, Masao Miyagawa, Teruhito Mochizuki

**Affiliations:** 1 grid.255464.40000000110113808Ehime University Graduate School of Medicine, Toon, Japan; 2 grid.459909.80000000406406159Saiseikai Matsuyama Hospital, Matsuyama, Japan

## Background

Feature-tracking cardiovascular magnetic resonance (FT-CMR) provides quantification of myocardial strain by analyzing cine MR images. A previous study has reported that CS measured by FT-CMR showed reasonable agreement with tagged MR in healthy volunteers. However, the usefulness of FT-CMR in patients with old myocardial infarction (OMI) has not been investigated. The purpose of this study was to evaluate diagnostic ability of CS by FT-CMR in patients with OMI.

## Methods

Between March 2011 and August 2012, a total of 20 consecutive patients with OMI were enrolled in this study. All cases were performed CMR examination using a 3-Tesla MR scanner (Philips Achieva). CS by FT-CMR was analyzed using Ziostation2 (Ziosoft Inc., Tokyo, Japan). The peak subendocardial CS was quantified for 16 segments of 3 short-axis slices (basal, mid, and apical). With interobserver consensus, myocardial segments were categorized as remote normal segments (n = 173), adjacent segments (n = 70), and infarcted segments (n = 77) from the results of late gadolinium enhancement (LGE) with CMR. An infarcted segment was defined as an area with the presence of LGE. An adjacent segment was defined as an area adjacent to infarcted segment.

## Results

The peak subendocardial CS was significantly lower in infarcted segments than in remote normal segments. (-6.3 ± 3.9 vs -11.8 ± 3.3; p < 0.001). Moreover, the peak subendocardial CS was significantly lower in adjacent segments than in remote normal segments. (-9.5 ± 3.7 vs -11.8 ± 3.3; p < 0.05). A cutoff value of -7.9% for peak subendocardial CS allowed differentiation between normal and infarcted segments with a sensitivity of 68%, specificity of 75%, accuracy of 73%, positive predictive value of 55%, negative predictive value of 84%, and an area under the curve (AUC) of 0.75.

## Conclusions

FT-CMR can quantify myocardial strain without increasing examination time. Moreover, FT-CMR is useful for detecting infarcted segments.

**Figure 1:**
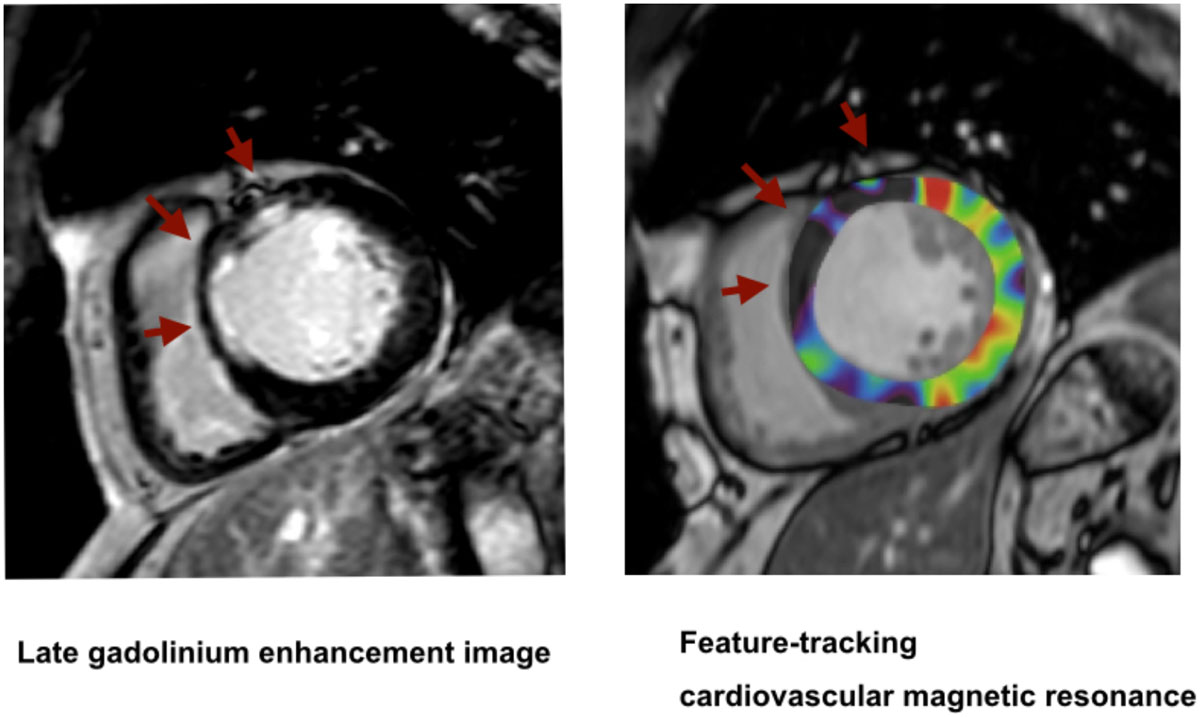
**A short-axis late gadolinium enhancement image of a basal slice shows enhancement in the anteroseptal region**. The peak subendocardial circumferential strain was decreased in anteroseptal region.

**Figure 2:**
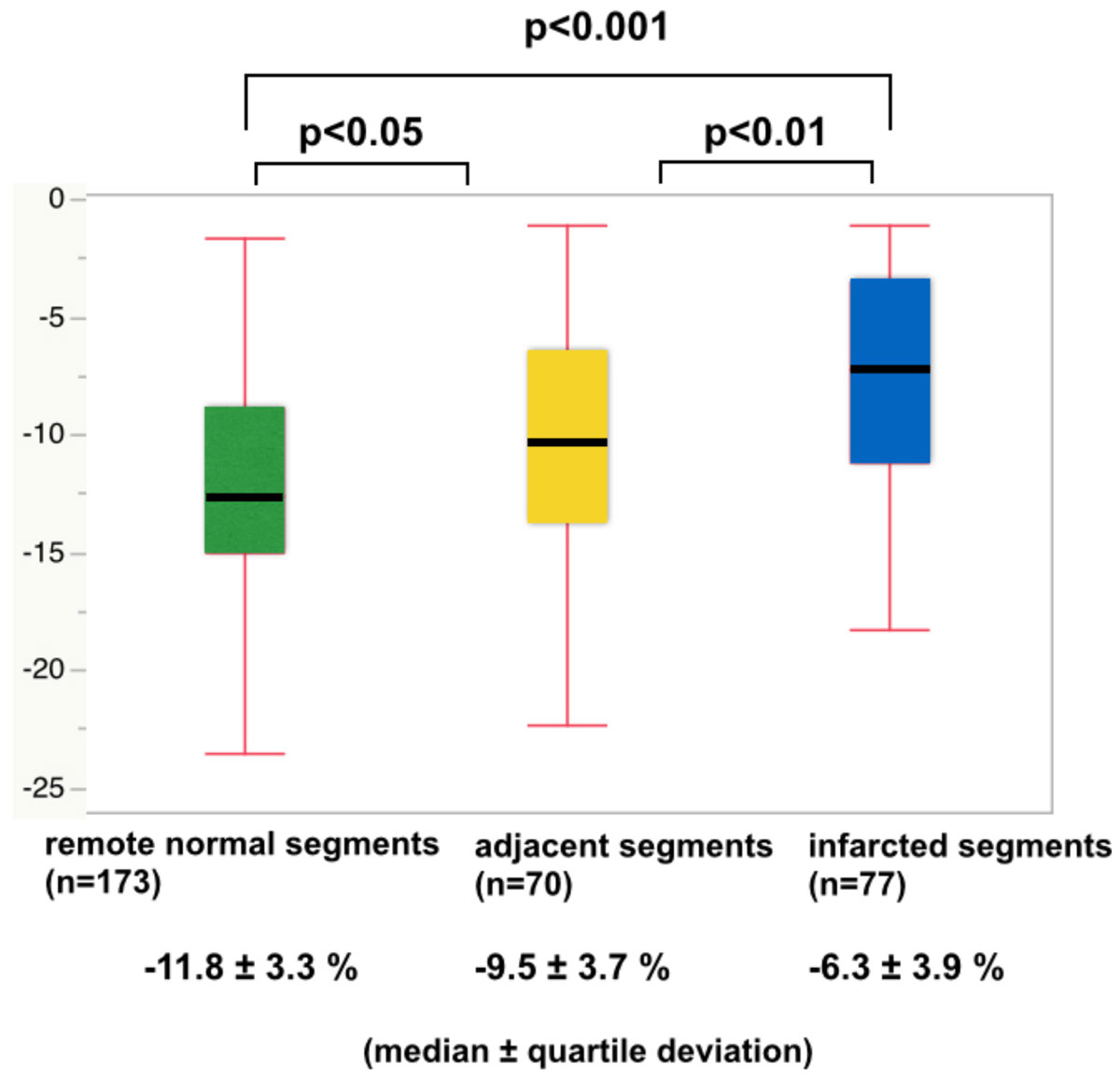
**The peak subendocardial circumferential strain (CS) of remote normal segments, adjacent segments, and normal segments**. The peak subendocardial CS was significantly lower in infarcted segments than in remote normal segments. Moreover, the peak subendocardial CS was significantly lower in adjacent segments than in remote normal segments.

